# Lack of Effectiveness of Antiretroviral Therapy in Preventing HIV Infection in Serodiscordant Couples in Uganda: An Observational Study

**DOI:** 10.1371/journal.pone.0132182

**Published:** 2015-07-14

**Authors:** Josephine Birungi, Jeong Eun Min, Katherine A. Muldoon, Pontiano Kaleebu, Rachel King, Sarah Khanakwa, Maureen Nyonyintono, YaLin Chen, Edward J. Mills, Fred Lyagoba, Manon Ragonnet-Cronin, Jonathan Wangisi, Lillian Lourenco, David M. Moore

**Affiliations:** 1 The AIDS Support Organisation, Kampala, Uganda; 2 BC Centre for Excellence in HIV/ AIDS, Vancouver, Canada; 3 University of British Columbia, Faculty of Medicine, Vancouver, Canada; 4 Medical Research Council (UK)-Uganda Virus Research Institute Research Unit on AIDS, Entebbe, Uganda; 5 London School of Hygiene and Tropical Medicine, London, United Kingdom; 6 University of California, San Francisco, Kampala, Uganda; 7 Faculty of Health Sciences, University of Ottawa, Ottawa, Canada; 8 Institute of Evolutionary Biology, University of Edinburgh, Edinburgh, United Kingdom; Infectious Disease Service, UNITED STATES

## Abstract

**Background:**

We examined the real-world effectiveness of ART as an HIV prevention tool among HIV serodiscordant couples in a programmatic setting in a low-income country.

**Methods:**

We enrolled individuals from HIV serodiscordant couples aged ≥18 years of age in Jinja, Uganda from June 2009 – June 2011. In one group of couples the HIV positive partner was receiving ART as they met clinical eligibility criteria (a CD4 cell count ≤250 cells/ μL or WHO Stage III/IV disease). In the second group the infected partner was not yet ART-eligible. We measured HIV incidence by testing the uninfected partner every three months. We conducted genetic linkage studies to determine the source of new infections in seroconverting participants.

**Results:**

A total of 586 couples were enrolled of which 249 (42%) of the HIV positive participants were receiving ART at enrollment, and an additional 99 (17%) initiated ART during the study. The median duration of follow-up was 1.5 years. We found 9 new infections among partners of participants who had been receiving ART for at least three months and 8 new infections in partners of participants who had not received ART or received it for less than three months, for incidence rates of 2.09 per 100 person-years (PYRs) and 2.30 per 100 PYRs, respectively. The incidence rate ratio for ART-use was 0.91 (95% confidence interval 0.31-2.70; p=0.999). The hazard ratio for HIV seroconversion associated with ART-use by the positive partner was 1.07 (95% CI 0.41-2.80). A total of 5/7 (71%) of the transmissions on ART and 6/7 (86%) of those not on ART were genetically linked.

**Conclusion:**

Overall HIV incidence was low in comparison to previous studies of serodiscordant couples. However, ART-use was not associated with a reduced risk of HIV transmission in this study.

## Introduction

Antiretroviral therapy (ART) has been shown to dramatically reduce plasma viral load (VL) in HIV-infected individuals. Studies conducted prior to the widespread introduction of ART in Uganda demonstrated that the HIV VL of the infected partner is the primary determinant of HIV transmission among serodiscordant couples [1] This and other studies led to research examining the effects that HIV treatment has contributed to reducing the spread of HIV in industrialized countries with large comprehensive, treatment programs[2, 3]. The HPTN 052 study confirmed, in a randomized trial, that the initiation of early ART is associated with a 96% reduction in HIV transmission between serodiscordant couples, many of whom were recruited in low and middle-income countries[4]. This research has led to further speculation that expansion of HIV treatment in sub-Saharan Africa may eventually lead to similar reductions in HIV transmission as observed in high-income countries [5]. Recently reported empirical evidence from South Africa appears to support this assertion [6].

However, the context of highly-resourced randomized trials and ecologic studies from industrialized countries is very different from that of ART programs in low-income countries, where the majority of HIV-infected individuals accessing treatment reside. In such programs support for adherence to therapy, transportation, nutrition and other ancillary services are often lacking. As well, human resources [7, 8] and clinical service infrastructure [9] to support ART delivery are also often limited. Therefore, there may be challenges in extrapolating the results of previous research to the realities of how and where most HIV-infected individuals receive care and treatment in resource-limited settings.

We conducted an observational study to examine the effectiveness of ART as prevention in a large HIV care and treatment program in rural Uganda in order to determine the real-world effectiveness of HIV treatment as prevention among serodiscordant couples.

## Methods

We conducted a prospective cohort study of co-habiting HIV serodiscordant couples of individuals aged ≥18 years of age known as the Highly Active Antiretroviral therapy as Prevention (HAARP) study. All HIV-infected participants were clients of The AIDS Support Organization (TASO), in Jinja, Uganda. Participants were eligible if they reported at least two episodes of sexual intercourse in the previous three months. Prior to enrollment, the HIV negative partner must have undergone an HIV test and received the results. Both the positive and negative participants were required to have already disclosed their HIV serostatus to their partner. The TASO clinic in Jinja provides care to over 8000 HIV infected individuals with over 5000 receiving ART. The ART program began in 2004 and is financed through the US Presidents Emergency Fund for AIDS Relief.

Study participants provided informed written consent to their participation. Prior to study enrollment a research assistant read aloud the consent form in the preferred, of one of three local languages and responded to all questions raised by potential recruits. If the individual agreed to study participation, they then signed the form (either with a signature, or with a thumb-print for individuals who could not write). The study, including the consent forms and data collection tools, received scientific and ethics approval from the Institutional Review Boards of the Uganda Virus Research Institute and the Uganda National Council for Science and Technology in Uganda and the University of British Columbia in Vancouver, Canada.

In one group of couples the HIV positive partner was receiving ART because of meeting the clinical or laboratory eligibility requirements in effect at the time of the study; a CD4 cell count ≤250 cells/ μL or WHO Stage III or IV disease[10]. In the second group of couples, the infected partner was not yet ART-eligible. Both groups received couple-based HIV transmission risk-reduction counseling and condoms on a quarterly basis. Clinical and behavioral data were collected every six months and the HIV-infected participants received their routine care through TASO clinicians every one to three months. Routine VL testing is not available for TASO clients, but clinical monitoring and CD4 cell count testing are conducted every six months to monitor ART effectiveness in accordance with the Ugandan guidelines.

HIV incidence in the two groups was measured by testing the uninfected partner every three months using two different point of care tests, applied in a serial algorithm (Determine, Alere Medical Co Ltd; StatPak, Chembio Diagnostic Systems Inc.). In the event of discordant results between the first and second tests, a third test (UniGold, Trinity Biotech Manufacturing Ltd.) was used to make the final diagnosis. All new HIV diagnoses were also subsequently confirmed by a laboratory-based ELISA test. The study was powered to detect a 75% reduction in HIV incidence associated with ART-use assuming that HIV incidence in the couples with untreated HIV would be 4 per 100 person years with an α of 0.05 and a power of 0.8, using a two-sided test.

For individuals who initiated ART near enrollment or during the study, observation time was considered to be not on ART until three months after the individual first started receiving ART. Once these individuals had received ART for three months, all subsequent observation time was considered to be while receiving ART. We collected sociodemographic and behavioural data through questionnaires administered by research assistants at enrollment and every six months thereafter. We also conducted clinical assessments at the same intervals and collected vaginal swabs for bacterial vaginosis and performed rapid diagnostic tests for syphilis (Determine, Inverness Medical, UK), every six months. Treatment for other sexually transmitted infections was based on syndromic management in accordance with local guidelines.

We collected and stored plasma and serum samples every six months for viral load testing (COBAS Ampliprep/TaqMan HIV-1 test V2.0 (Roche, Mannheim, Germany)) and herpes simplex type 2 (HSV2) antibody testing (Kalon HSV2 IgG ELISA, Guilford, UK) at the end of the study. In couples where HIV seroconversion occurred, we conducted VL testing on all samples from the HIV positive participant during the study and for non-seroconverting couples, we tested only the sample drawn at the last study visit. We conducted HSV2 serology on all study participants using the enrollment sample and again on those individuals who were HSV2 negative at enrollment on the last samples drawn. HIV sequencing was conducted on plasma samples drawn from the HIV positive participant and their partners who seroconverted where the VL measured was ≥300 copies/ mL using an in house method. For seroconverting couples where we could not obtain sufficient virus in plasma samples for genotyping, we drew additional whole blood samples to extract proviral DNA from intact CD4 cells.

Briefly, RT-PCR followed by nested PCR of the 5’ region of the *pol* gene generated a 1341 base-pair fragment. We analyzed Protease (PR aa 1–99) and Reverse Transcriptase (RT aa 1–320) regions of the *pol* gene (fragment length 1341 base-pairs). This fragment was purified, sequenced using the BigDye Terminator v3.1 Cycle Sequencing Kit (Applied Biosystems International, Foster City, CA), and analysed on an ABI Prism 3130 Genetic Analyser (Applied Biosystems International). All resulting sequences were genotyped using SCUEAL [11]. In order to conduct a phylogenetic analysis with appropriate community controls, all Ugandan HIV *pol* sequences with dates were downloaded from the Los Alamos National Laboratory Database (HXB2 positions 2253 to 3549, min length 600 bases). A sequence similarity search (ViroBlast) [12]was performed on each subtype dataset separately to retrieve the ten closest sequences to each of the study sequences within the LANL Ugandan dataset. Phylogenetic trees for subtypes A1, D and A1D recombinants were reconstructed using Bayesian Evolutionary Analysis by Sampling Trees (BEAST)[13]. Analyses were run in duplicate under a Skyride model with a gamma distributed uncorrelated lognormal molecular clock and SRD06 substitution model, for 100,000,000 generations. Convergence was assessed in Tracer and duplicate runs were combined. Maximum clade credibility (MCC) trees for genetic distance and time were summarized with Tree Annotator. For each couple pair, we generated Time to Most Recent Common Ancestors and pairwise genetic distances from MCC trees.

We conducted Kruskal Wallis and Chi-squared tests to compare couples where the HIV positive participant did not receive ART, with those where the positive participant was receiving ART from enrollment and couples where the positive participant began ART during the study. We also conducted bivariate analyses comparing couples where seroconversion occurred during follow-up with those where seroconversion did not occur. We calculated the incidence rate ratio for HIV seroconversion using exact Poisson regression and used univariate Cox proportional hazards modeling to examine the hazard associated with time-to-seroconversion in the HIV negative participants, with ART exposure of the HIV positive participant used as a time-updated variable. Given the small number of transmission events we observed, we were unable to develop multivariate models, rather we conducted several stratified Cox proportional hazard analyses[14] to account for different baseline hazard function for each of several potential confounders. These analyses stratified participants based on: 1) the circumcision status the male participants, 2) the HSV2 serostatus of the HIV infected participant 3) the HSV2 serostatus of the HIV uninfected participant, 4) the gender of the HIV-infected partner and 5) the recorded CD4 cell count nadir of the HIV positive participant. All analyses were conducted using SAS Version 9.3 (SAS Corporation, Cary, North Carolina).

## Results

A total of 586 couples were enrolled between June 1, 2009 and June 20, 2011. In 249(42.5%) of these couples, the HIV-infected participant was receiving ART at enrollment and in 99 (17%) couples the HIV positive participant initiated ART during the study. In the remaining 238 (40.6%) couples the HIV-infected participant did not receive ART during the study. The median duration of ART-use for those on ART at enrollment was 2.3 years. Table 1 shows the comparison of these three groups of participants. The gender distribution of the HIV-infected partner was somewhat uneven through the three groups, but these differences were not statistically significant (p = 0.074). We found a lower proportion of circumcised male partners among the couples receiving ART at enrollment (34% vs.45% vs. 43%), but these differences were only marginally statistically significant (p = 0.053). There were no differences in terms of proportion of participants in polygyneous relationships, intergenerational partnerships, number of children or other sociodemographic variables.

**Table 1:** Characteristics of individuals in 586 discordant couples enrolled in the HAARP Study.

		Never on ART during the study	Began ART during the study	On ART at enrollment	P- value
**N (%)**		238 (41%)	99 (17%)	249 (42%)	
**Gender of HIV positive participant**	Male (%)	121 (51%)	60 (61%)	150 (60%)	0.074
	Female (%)	117 (49%)	39 (39%)	99 (40%)	
**Age of male partner** Median (IQR)		40 (34–47)	41 (36–50)	43 (37–50)	0.002
**Age of female partner** Median (IQR)		33 (29–40)	36 (30–40)	36 (30–40)	0.002
**Intergenerational partnership (>10 yrs age difference between male and female partner)**		83 (35%)	29 (29%)	90 (36%)	0.472
*Primary Language Spoken (male response)	Lusoga	164 (69%)	63 (65%)	144 (60%)	0.095
	Other	72(31%)	34 (35%)	96 (40%)	
**Polygyneous partnership (%)**		58 (24%)	26 (26%)	61 (25%)	0.928
**Male partner circumcised (%)**		102 (43%)	44 (45%)	83 (34%)	0.053
**Used a condom at last sex (%) (positive partner)**		159 (67%)	68 (69%)	188 (76%)	0.095
**Used a condom at last sex (%) (negative partner)**		164 (69%)	69 (70%)	192 (77%)	0.101
**Condom use in the last 3 months (male response)**	Always	138 (58%)	61 (62%)	167 (67%)	0.003
	Sometimes	51 (21%)	15 (15%)	58 (23%)	
	Never	49 (21%)	23 (23%)	24 (10%)	
**Age of sexual debut (female)** Median (IQR)		16 (15–18)	16 (15–18)	16 (15–18)	0.191
**# of lifetime sex partners (male)** Median (IQR)		6 (4–12)	6 (4–10)	7 (4–13)	0.315
**# of lifetime sex partners (female)** Median (IQR)		3 (2–4)	3 (2–4)	3 (2–4)	0.573
**Male sexual decision making (male)**		90 (38%)	35 (36%)	87 (35%)	0.819
**Duration (years) of relationship with primary partner (positive partner with partner 1)** Median (IQR)		10 (5–18)	12 (6–22)	12 (6–21)	0.018
**Number of sexual partners for non-polygynous males**	More than one	9 (5%)	5 (7%)	9 (5%)	0.786
	One	171 (95%)	68 (93%)	179 (95%)	
**>1 sexual partner reported by female**		4 (2%)	1 (1%)	3 (1%)	0.896
**Intend to have more children (male response)**		88 (41%)	34 (38%)	77 (35%)	0.401
**Intend to have more children (female response)**		51 (26%)	25 (30%)	47 (24%)	0.605

Couples where the positive participant was receiving ART at enrollment were more likely to report always using condoms in the past three months in comparison to those who initiated during the study or were never on ART (67% vs. 62% vs.58% respectively; p = 0.003). They also reported longer relationships with their primary sexual partner than non-ART couples (median 12 vs. 10 years; p = 0.018), but not longer than couples who started ART during the study (median 12 years). There were no differences between the three groups in terms of condom use at last sex, pregnancy intentions, alcohol use, number of non-spousal sexual partners or other behavioural characteristics.

There were also differences in clinical characteristics between the three groups (Table 2). The HIV positive partners in the couples receiving ART at enrollment or began during the study were more likely to be HSV2 seropositive (91% and 92%, respectively) in comparison to those who were never on ART during the study (84%) (p = 0.050). There were no differences in the use of injectable contraception, genital ulcer disease or bacterial vaginosis across the groups.

**Table 2:** Clinical characteristics of study participants.

		Never on ART during the study	Began ART during the study	On ART at enrollment	P- value
**CD4 cell count at enrollment of HIV positive partner** Median (IQR)		515 (389–684)	248 (156–366)	392 (240–539)	<0.001
**Median VL (log 10 copies/ mL) at study exit or prior to sero-conversion**		4.4 (3.5–4.9)	1.4 (1.3–2.7)	1.3 (1.3–1.5)	<0.001
**VL >1000 copies/ mL at study exit or prior to seroconversion** *		140/177 (79%)	23/98 (23.%)	16/216 (7%)	<0.001
**HSV2 serology of HIV negative partner**	HSV2 positive at enrollment	183 (77%)	84 (87%)	203 (83%)	0.067
	Incident HSV2 infection	2 (1%)	2 (2%)	5(2%)	
	HSV2 negative	53 (23%)	11 (11%)	37 (17%)	
**HSV2 serology of HIV positive partner**	HSV2 positive at enrollment	197 (84%)	91 (92%)	222 (91%)	0.050
	Incident HSV2 infection	2 (1%)	1 (1%)	0 (0)	
	HSV2 negative	35 (15%)	7 (7%)	23 (9%)	
**Any bacterial vaginoisis during study**		44 (19%)	17 (17%)	39 (16%)	0.709
**Any bacterial vaginoisis among HIV negatives during the study**		17 (14%)	8 (13%)	22 (15%)	0.962
**Use Other Family Planning (HIV positive partner’s response)**		29 (12%)	12 (13%)	25 (10%)	0.711
**Use injectable contraception (female response)**		144 (61%)	67 (68%)	163 (66%)	0.371

*Three of the seroconverter couples did not have VL results prior to seroconversion, for two of these couples we used the most recent VL result which occurred after seroconversion

**Fig 1:**
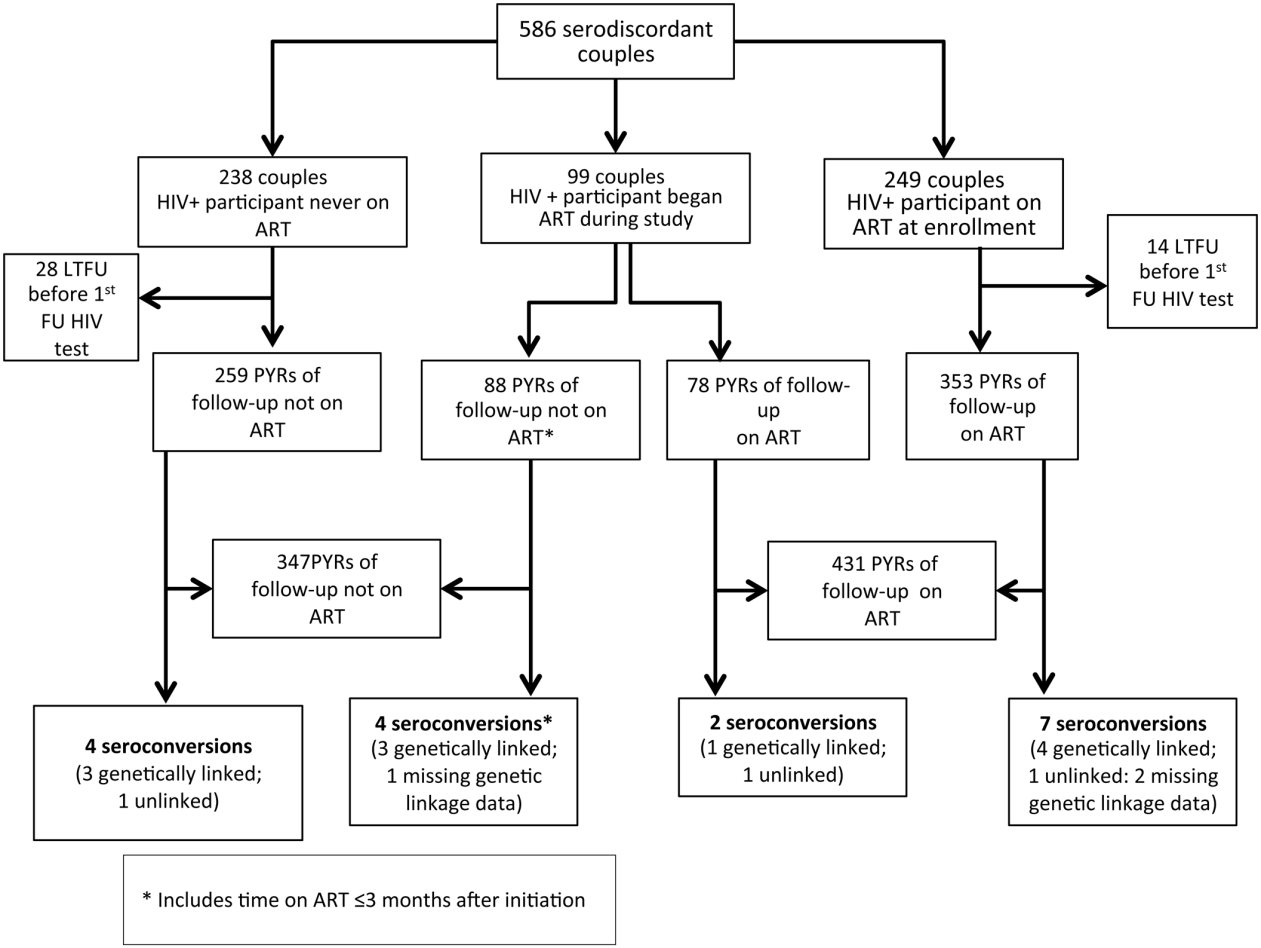
Study profile of the Highly Active Antiretroviral therapy as Prevention (HAARP) Study.

Follow-up of study participants continued until December 14, 2011 when the median duration of study participation was 1.5 years. A total of 42 couples did not return for the first follow-up visit, such that 544 couples were able to be assessed for an outcome (Fig 1). We found 9 new infections among partners of participants who had been receiving ART for at least three months and 8 new infections in partners of participants who had not received ART or received it for less than three months, for incidence rates of 2.09 per 100 person-years (PYRs) and 2.30 per 100 PYRs, respectively. The incidence rate ratio was 0.91 (95% confidence interval [CI] 0.31–2.70; p = 0.999) associated with ART-use by the positive partner. Among the 99 couples where the positive partner began ART during the study, the incidence rate was 2.56 per 100 PYRs for the time observed on ART and 4.54 per 100 PYRs for the observation time not on ART, for an IRR of 0.56 (95%CI: 0.10–3.08; p = 0.804). A total of 3/8 ART participants and 8/8 of the non-ART participants had VLs>1000 copies/ mL either immediately prior to- or just after seroconversion. One of the ART participants did not have a measured VL result during the study. We were able to determine partial viral *pol* gene sequence from both partner viruses in 14 of the 17 seroconverter couples. Of these 79% (11/14) were found to be genetically linked; 5/7(71%) in the ART arm and 6/ 7(86%) in the non-ART arm. Linked infections were always supported by posterior probabilities of 1. Mean pairwise genetic distance among couples was 0.02 nucleotide substitutions per site, and median 0.015.

Table 3 shows the characteristics of the couples where the HIV positive participant was receiving ART prior to seroconversion of their partner. Note that only three of the HIV positive individuals who were receiving ART whose partners seroconverted had VLs>1000 copies/ mL, although for one ART participant, we were missing VL results. One of these three self-reported clear non-adherence to therapy, was found to have a VL of >300,000 and did not consistently use condoms. Another individual initiated treatment just over three months prior to his partner’s seroconversion and had a previous VL off treatment was >250,000 copies/ mL, but a VL taken on the day of his partner’s first positive test of 176 copies/ mL. For all other seroconversions occurring among couples where the positive partner was receiving ART, the measured VL prior to seroconversion was <1000 copies/ mL. Four participants seroconverted after their partner’s most recent viral load was <20 copies/ mL; two of these were genetically linked, one was unlinked and one did not have linkage data available. In one of the nine seroconverting participants from ART couples, we found evidence of transmitted drug resistance but no evidence of resistance mutations in the viruses of their HIV positive partner. This is most likely due to the fact that the latter samples came from pro-viral DNA samples and not from plasma.

**Table 3:** Characteristics of couples where seroconversion occurred when HIV participant was receiving ART for more than 3 months.

Sex of seroconverter	Age	VL before seroconversion of HIV + Partner (copies/mL)	Resistance mutations in seroconverter virus	Resistance Mutations in HIV positive ppt virus	HIV + ppt on ART at enrollment	ART Duration prior to sero-conversion	Adherent to therapy ^(^ ^1^ ^)^	Consistent condom-use ^(^ ^2^ ^)^	Genetically linked transmission	ART regimen prior to sero-conversion
F	29	233	None	M184V K103N	Yes	6 months	Yes	Yes	YES	AZT, 3TC, EFV
M	47	Not detected	None	D67G, M184V, V106A, F227L	Yes	9 months	Yes	Yes	YES	AZT, 3TC, NVP
F	35	3704	M184V, T215Y, K103N, V179T	None	Yes	25 months	Yes	Yes	YES	AZT, 3TC, NVP
F	45	No result	No result	No result	Yes	36 months	Yes	Yes	No result	AZT, 3TC, NVP
F	45	Not detected	None	None	Yes	65 months	Yes	No	YES	AZT, 3TC, NVP
F	36	235,789 ^(^ ^3^ ^)^	None	None	No	3 months	Yes	Yes	YES	AZT, 3TC, EFV
M	32	317,773	No result	No result	Yes	29 months	No	No	No result	AZT, 3TC, NVP
F	22	Not detected	None	None	Yes	13 months	Yes	Yes	NO	AZT, 3TC, EFV
F	37	<20	None	None	No	15 months	Yes	Yes	NO	AZT, 3TC, NVP

Notes:

^1^ Adherence is a composite of two questions: “How often do you use miss taking your ARVs?” and “How many pills have you missed in the last week?”. If participant reported any response other than “never” and “0”, respectively, was reported as non-adherent

^2^ Condom use is a composite of two questions: “How often do you use condoms?” and “Did you use a condom the last time you had sex?”. If participant reported any response other than “always” and “yes”, respectively, condom use is reported as inconsistent

^3^ VL result taken on the day of serconversion = 176 copies/ mL. Reported value of 235,789 copies/mL was prior to ART initiation, 6 months prior to seroconversion.

The bivariate analysis of factors associated with seroconversion is shown in Table 4. Participants who were in polygynous relationships (47% vs. 24%; p = 0.042); couples where the female participant reported an early age of sexual debut (median age 15 years vs. 16 years; p = 0.018) and couples where the male participant reported that he decides when they have sex (59% vs. 35%; p = 0.044) were associated with seroconversion during the study. No association was found with HSV2 seropositivity during the study and HIV seroconversion. Having a VL>1000 copies/ mL was also associated with seroconversion (69% vs. 35%; p = 0.006). However, ART- use for greater than three months was not associated (p = 0.550). A larger proportion of women were the HIV negative participant in the couples where seroconversion occurred (13/17 [76%] vs. 293/527 [56%]). However, this was not statistically significant (p = 0.088).

**Table 4:** Bivariate Analysis comparing 17 discordant couples where seroconversion occurred with 527 discordant couples where no seroconversion occurred.

		Seroconverters (n = 17)	Non seroconverters (n = 527)	P- value
**ART status (%)**	Never on ART	4 (24%)	206 (39%)	0.145
	Began ART during the study	6 (35%)	93 (18%)	
	On ART at enrollment	7 (41%)	228 (43%)	
**ART status (%)**	On ART at least 3 months	9 (53%)	317 (60%)	0.550
	Not on ART or on ART ≤3 months	8 (47%)	210 (40%)	
**Sex of HIV negative participant (%)**	Female	13 (76%)	293 (56%)	0.088
	Male	4 (24%)	234 (44%)	
**Intergenerational relationship (>10 yrs age difference between male and female partner) (%)**		7 (41%)	183 (35%)	0.583
**Primary Language Spoken (male response) (%)**	Lusoga	12 (71%)	335 (65%)	0.645
	Other	5 (29%)	179 (35%)	
**Polygyneous partnership (%)**		8 (47%)	126 (24%)	0.042
**Male partner circumcised (%)**		4 (24%)	210 (40%)	0.163
**Used a condom at last sex (positive partner) (%)**		13 (76%)	374 (71%)	0.789
**Used a condom at last sex (negative partner) (%)**		13 (76%)	388 (74%)	1.000
**Condom use in the last 3 months (male) (%)**	Always	12 (71%)	337 (64%)	0.937
	Sometimes	3 (18%)	101 (19%)	
	Never	2 (12%)	89 (17%)	
**Age of sexual debut (male)** Median (IQR)		19 (15–23)	18 (16–20)	0.314
**Age of sexual debut (female)** Median (IQR)		15 (14–16)	16 (15–18)	0.018
**# of lifetime sex partners (male)** Median (IQR)		4 (4–10)	6 (4–12)	0.261
**# of lifetime sex partners (female)** Median (IQR)		4 (3–7)	3(2–4)	0.085
**Male sexual decision making (male response) (%)**		10 (59%)	184 (35%)	0.044
**Duration of relationship with primary partner (in years)** Median (IQR)		7.8 (4.5–12.5)	11.6 (5.8–20.2)	0.163
**Number of sexual partners for non-polygynous males (%)**	More than one	0 (0%)	22 (5%)	1.000
	One	9 (100%)	379 (95%)	
**>1 sexual partner reported by female**		0	8 (2%)	1.000
**Intend to have more children (male response)**		5 (42%)	172 (36%)	0.765
**Intend to have more children (female response)**		6 (40%)	103 (24%)	0.220
**Median VL (log 10 copies/ mL) at study exit or prior to sero-conversion**		4.8 (1.8–5.15)	1.5 (1.3–4.2)	0.006
**VL >1000 copies/ mLat study exit or prior to sero-conversion (n and %)**		11/16 (69%)	168/475 (35%)	0.006
**CD4 cell count at enrollment of HIV positive partner** Median (IQR)		329 (162–422)	419 (274–592)	0.079
**HSV2 serology at enrollment of HIV negative partner (n and %)**	HSV2 positive	13 (93%)	425 (81%)	0.592
	HSV2 negative	1 (7%)	108 (19%)	
**HSV2 serology at enrollment of HIV positive partner (n and %)**	HSV2 positive	14 (88%)	460 (88%)	0.725
	HSV2 negative	2 (13%)	61 (12%)	
**Any genital ulcer disease reported by HIV positive**		1 (6%)	9 (2%)	0.276
**Any genital ulcer disease reported by HIV negative**		0 (0%)	3 (1%)	1.000
**Any bacterial vaginosis during study**		2 (12%)	92 (17%)	0.750
**Any bacterial vaginosis among HIV negatives during the study**		1 (8%)	44 (15%)	0.700
**Use Other Family Planning (HIV positive partner’s response)**		0	61 (12%)	0.240
**Use injectable contraception (female response)**		12 (71%)	339 (64%)	0.602

*Three of the seroconverter couples did not have VL results prior to seroconversion, for two of these couples we used the most recent VL result which occurred after seroconversions

Univariate Cox proportional hazards modeling found that no association with time-updated ART use (hazard ratio [HR] 1.07; 95% confidence interval [CI] 0.41–2.80). Similarly none of our stratified Cox analyese demonstrated associations between ART use and seroconversion. The HR for ART-use when stratified by male circumcision status was 1.01 (95% CI 0.39–2.67); when stratified by HSV2 serostatus of the HIV positive participant it was 0.94 (95% CI 0.35–2.55); when stratified by the HSV2 serostatus of the HIV negative participant it was 0.90 (95% CI 0.31–2.60); when stratified by the gender of the HIV positive participant it was 1.02 (95% CI 0.39–2.68) and when stratified by the CD4 cell count nadir of the HIV positive ≤200 cells/μL, it was 0.581 (95% CI 0.10–3.27).

## Discussion

In this study, ART use by the HIV positive partner was not associated with a reduced risk of HIV transmission among HIV serodiscordant couples. This result contrasts with many previous clinical studies of ART as prevention that have found effectiveness varying from 26%- 96% [4, 15–19], as well as several ecologic studies[2, 3]. Both the median log10 VL and the proportion of participants with VLs>1000 copies/ mL were significantly higher among the HIV positive partners in the seroconverting couples, but this did not directly correlate with a reduced risk of transmission from the ART couples. Participants in polygyneous relationships and where the male partner made sexual decisions for the couple were more likely to seroconvert during our study, as were couples where the female participant reported an early age of sexual debut.

The HPTN 052 study found a 96% reduction in HIV transmission associated with the early use of ART among 1763 serodiscordant couples [4]. However, the estimate of effectiveness was reduced to 75% if non-genetically linked transmissions were included. Another observational study from multiple sites in Eastern and Southern Africa found a similar 92% reduction in transmission risk associated with ART use[15]. However, the context of ART-use in our study was quite different than in these, in that most participants who were receiving ART had done so for more than two years before study enrollment and they did not have access to VL testing. Nevertheless, virologic control among the HIV-infected participants was very good in that only 7% of the individuals who were receiving ART at initiation and 23% of individuals who initiated ART during the study had a VL measurement >1000 copies/ mL, so that large numbers of ART patients with undiagnosed treatment failure cannot provide an explanation for these results. A Cochrane review examining the published literature on ART for prevention of HIV transmission among serodiscordant couples, found an overall incidence rate ratio of 0.34 (95% CI 0.13–0.92) among seven previously published observational studies with substantial heterogeneity between studies[20]. When the analysis was restricted to only studies from low-income countries a similar incidence rate ratio was noted (0.27), but the result was only marginally statistically significant (p = 0.06). The authors noted that the quality of the evidence was low for this sub-analysis, an area to which our current study may now contribute.

The relatively low rate of HIV incidence in the non-ART couples (2.30 per 100 PYRs) may be due to the high prevalence of condom-use in this cohort, but may more accurately reflect the background HIV transmission risk of serodiscordant couples where the positive partner is already engaged in HIV care and receiving prevention counselling. In the systematic review, above, the median incidence rate for this group was 7.5 per 100 PYRs[20]. Reported condom-use at last sex in our study was over 70% at enrollment and increased to over 90% by one year of follow-up[21]. In fact, it appears from our sub-analysis of couples where the HIV positive participant began ART during the study, that ART-use may have prevented some HIV transmission events, since the incidence rate for the individuals starting treatment was much higher (4.54 per 100 PYRs) than in the period immediately following treatment initiation (2.56 per 100 PYRs), although these differences were not statistically significant. The overall transmission rate of 2.03 per 100 PYRs among the partners of HIV infected participants who were receiving ART at any point during our study approximates the mid-point estimate (2.5 per 100 PYRs) derived from systematic review[20]. Our findings suggest that efforts to promote the widespread adoption of other HIV prevention strategies, such as condom promotion, couples counselling and testing, partner reduction and medical male circumcision, should continue in parallel with the ongoing expansion of ART.

It is possible that the higher proportion of uncircumcised men and HSV2 seroprevalence in the couples where ART was used may have diminished the effectiveness of ART in preventing transmission. However, these differences in distribution were quite small (9% for circumcision, 8% for HSV2 prevalence among HIV positives) and seem unlikely to completely counteract the expected efficacy of ART in preventing HIV transmission. Furthermore, we did not find any effect on ART-use by the HIV positive partner in analyses stratified on the basis of circumcision status, gender or HSV2 seroprevalence. Nevertheless, we cannot rule out interactions in the differences between these important co-factors of HIV transmission. However, it is also worth noting that some of the differences in variables known to be associated with HIV transmission, such as condom-use and relationship duration, would have biased our study towards having a reduced risk of HIV transmission among the ART couples, irrespective of the effect of ART. Such factors have been previously identified as biases in observational studies examining this issue[22].

Our study has a number of limitations. Firstly, our study was underpowered to detect the moderately sized decreases in HIV transmission (75%) associated with ART-use because the incidence in the couples where the positive partner did not receive treatment was lower than we had anticipated. However, a post-hoc power calculation did find that we had sufficient statistical power to detect a 95% reduction in transmission associated with ART-use. Our study, however, cannot rule out an effect of ART which was less than this. Secondly, the VL measurements we obtained could have been taken up to six months prior to the date of seroconversion and therefore may have missed episodes of high viremia which may have caused transmission. The small number of transmission events also meant that we were unable to adjust our analyses to control for all potential confounders. Finally, as with all observational studies, confounding by other factors which we did not measure may also have contributed to the lack of effect we observed.

In summary, we did not demonstrate a benefit of the use of ART in preventing HIV transmission among serodiscordant couples in Uganda. A possible explanation for this observation is that couples were already using condoms and had participated in some risk reduction counseling, thus limiting the possibility to show further benefits from ART. Our results highlight the need for conducting operational research in similar program settings to determine the real-world impact of interventions which have been proven in clinical trials.
